# The Interaction of the IFN*γ*/JAK/STAT1 and JAK/STAT3 Signalling Pathways in EGFR-Mutated Lung Adenocarcinoma Cells

**DOI:** 10.1155/2022/9016296

**Published:** 2022-09-21

**Authors:** Yaguang Han, Yan Zhang, Ying Tian, Miao Zhang, Cheng Xiang, Qiang Zhen, Jiabao Liu, Yanhong Shang, Yiyi Zhao, Huarui Si, Aixia Sui

**Affiliations:** ^1^Department of Oncology, Shijiazhuang People's Hospital, Shijiazhuang 050030, China; ^2^HeBei Reproductive Hospital, Shijiazhuang 050000, China; ^3^Department of Thoracic Surgery, Shijiazhuang People's Hospital, Shijiazhuang 050000, China; ^4^Department of Oncology, Affiliated Hospital of Hebei University, Baoding 071000, China; ^5^Department of Oncology, Hebei General Hospital, Shijiazhuang 050051, China

## Abstract

**Purpose:**

It was reported that the EGFR (epidermal growth factor receptor) mutation status was related to primary immune resistance in NSCLC (non-small-cell lung cancer). ICIs (immune checkpoint inhibitors) have poor efficacy and large side effects for people with EGFR mutation. EGFR mutation was considered as a sign of immune therapeutic resistance, but its underlying mechanism is difficult to be determined. Combined with our research basis, we tried to explore the possible mechanism of primary drug resistance in EFGR mutant lung adenocarcinoma through the interaction between the JAK/STAT1 and JAK/STAT3 pathway.

**Materials and Methods:**

Cell apoptosis and viability test were used to study the role of the JAK/STAT signalling pathway in lung adenocarcinoma cell survival. Western blot, RT-PCR, and flow cytometry were employed to explore the changes of expression in JAK1/2, STAT1/3, PD-L1, and related signal molecules in the case of activation or inhibition of the JAK/STAT3 signalling pathway.

**Results:**

With inhibition of inhibiting the JAK/STAT3 signalling pathway by STAT3 inhibitors, we found IFN*γ*-JAK-STAT1 pathway activation by IFN*γ* could further keep lung adenocarcinoma cells from proliferation and promote its apoptosis. The inhibition of the JAK/STAT3 pathway results in the upregulation of JAK1/2, STAT1, IRF1, IRF9, and PD-L1 and downregulation of STAT3 and SOCS1.

**Conclusions:**

The absence of the IFN*γ*-JAK-STAT1 signal pathway is one of the main mechanisms for the ICI endogenous resistance. The abnormal activation of the downstream JAK/STAT3 pathway in cells with EGFR mutation may have antagonistic effects on the STAT1 induced antitumor immune response, which may cause the IFN*γ*-JAK-STAT1 pathway to lose its function. The mechanism may result in production of the immune tolerance of the EGFR mutant, which promotes immune escape.

## 1. Introduction

As one of the most common cancers, lung cancer has the highest morbidity and mortality worldwide [1, 2]. Lung cancer is divided into small cell lung cancer (SCLC), which account for 20% of cases, and non-small cell lung cancers (NSCLC), which account for 80% [3]. The optimal treatment period is often missed when patients are diagnosed with NSCLC. Seventy-five percent of NSCLC patients are diagnosed at the advanced stage, leading to a 5-year survival rate of less than 15%. Intrinsic resistance and acquired resistance present a major challenge in the treatment of NSCLC and they contribute to tumor progression and recurrence [4]. Over the past few decades, despite the rapid development of medical technology, the incidence and mortality of lung cancer has always been the highest among all types of cancer worldwide, almost becoming one of the most serious threat to human health [5]. The EGFR gene is an important oncogene associated with NSCLC. Previous statistics show that EGFR mutation happens in up to 47.9% of Asian NSCLC patients [6, 7], EGFR tyrosine kinase inhibitors (EGFR-TKIs) have become the first line of choice for patients with advanced EGFR mutation-positive NSCLC. However, most patients acquire resistance during treatment, and limited strategies to overcome EGFR-TKIs resistance are available.

In recent years, the clinical oncology landscape has been transformed by the immunotherapy, which has improved long-term survival for some patients. Immune checkpoint inhibitors (ICIs) have several benefits for NSCLC patients: excellent survival with long-term efficacy and reduced toxicity [8–12]. However, recent studies data show that the application of ICIs to EGFR mutant population has poor efficacy and side effects, and some secondary T790M mutants even had explosive progression. Therefore, EGFR mutation was once identified as a marker of immune resistance, but the mechanism is difficult to determine [13, 14]. However, the latest study of IMpower 150 published in 2018 unexpectedly brought new information to these patients. Subgroup analysis showed that the combination of anti-PD-L1 and chemotherapy brought unprecedented survival benefits to the previously identified “cold tumor” [15]. This study may open a window of hope for immunotherapy in people with EGFR mutations. This result suggests that the so-called “immune resistance” in people with EGFR mutations is not absolute, but completely reversible [16].

Current studies have shown that the absence of the IFN*γ* signalling pathway is one of the main mechanisms leading to endogenous drug resistance in ICIs [17]. IFN*γ*, produced by T cells, recognizes the corresponding receptors on tumor cells or antigen-presenting cells, producing antitumor response. IFN*γ* also stimulates major histocompatibility complex (MHC) molecules to recruit other immune cells. Therefore, mutations and deletions of proteins related to this pathway on tumor cells, such as JAK1 and JAK2, STAT1, and IRF1, the IFN*γ* receptor chain, can lead to resistance to immune checkpoint inhibitors [18].

EGFR mutations cause abnormal activation of the downstream JAK/STAT3 signalling pathway, leading to the expression of increased phosphorylated STAT3, and hence abnormal tumor proliferation, angiogenesis, invasion, and metastasis. Current studies have confirmed that STAT3-mediated inhibitory tumor immune response has an antagonistic effect on STAT1-driven antitumor immune response [19], which may lead to the loss of function of the IFN*γ*/JAK/STAT1 pathway. In this study, we verified that activation of the downstream JAK/STAT3 signalling pathway during EGFR mutation resulted in upregulation of the phosphorylated STAT3 expression, antagonized the expression of STAT1, and made the IFN*γ*/JAK/STAT1 signalling pathway where it was located play an abnormal role, which ultimately led to the generation of antitumor immune tolerance in patients with EGFR mutation and promoted immune escape. If this hypothesis is true, it will provide an important theoretical basis and clinical guidance for overcoming immune resistance in patients with EGFR mutation. It will also be of great significance for the treatment of these patients in the future.

## 2. Materials and Methods

### 2.1. Cell Lines and Cell Culture

Human NSCLC cell lines H1299 (EGFR-WT), PC-9 (EGFR-19del), H1975 (EGFR-T790M and L858R combined mutation), and H3255 (EGFR-L858R mutation) were obtained from the ATCC. Cells were cultured by RPMI 1640 medium (Corning, USA) with 15% fetal bovine serum (Corning, USA) and 1% penicillin/streptomycin mixture. The cells were cultured in a humidified incubator at 37°C containing 5% CO_2_. After treatment with IFN*γ* (Peprotech, USA) and/or STAT3 inhibitor (Cryptotanshinone Cayman, USA), cells were collected for analysis.

### 2.2. CCK-8 Assay

CCK-8 (Zoman Biotechnology, Beijing, China) was used to assess the viability of H1299, PC9, H1975, and H3255 cells. The cells were cultured in 96-well plates (5 × 10^3^ cells/well) in the incubator for 24 hours. Cells were then exposed to IFN*γ* (500 ng/ml), STAT3 inhibitor (6.74 *μ*mol), and combine IFN*γ* (500 ng/ml) with a STAT3 inhibitor (6.74 *μ*mol/L) for 24 hours. After treatment, the CCK-8 reagent (10 *μ*l) was dripped to the well and cells were cultured in the incubator for four hours. Absorbance was assessed using a microplate reader.

### 2.3. Flow Cytometry

FITC Annexin V/PI double staining to detect cell apoptosis rate, collecting cells in 15 ml centrifuge tube, centrifugal, 4°C precooling PBS wash twice, centrifugal supernatant, use the 1x Binding Buffer 100 mu l heavy suspension cells, and cell suspension to flow in the pipe, according to the grouping respectively to join 5 mu l FITC Annexin V and 5 mu PI l dye blending, at room temperature away from light incubation 15 min after add 400 uL 1x binding buffer blending, Cell apoptosis was detected by flow cytometry. The expression of PD-L1 on the cell surface was detected by flow cytometry, and the specific method was described in the instructions.

### 2.4. Cell Cycle Analysis

Cells were collected; 1.5 ml of precooled PBS and 3.5 ml of anhydrous ethanol in vortex state were added and mixed, and fix at 4°C for 30 min. Ethanol is removed from the cells by centrifugation, fixed, and then stained with 50 *μ*g/mL propidium iodide solution (BD Biosciences, USA) in the dark for 30 minutes. After 30 minutes of incubation, a BD flow cytometer was used to analyse the samples and the data were analysed by FlowJo software.

### 2.5. Western Blot

The samples were added with RIPA lysate, after homogenization, lysis, and centrifugation, the total protein extract was obtained. Concentration of the total protein in the extract was determined by BCA protein quantitative protocol. Protein electrophoresis was conducted by SDS-PAGE, followed by a constant current for 1 h to transfer the protein onto the PVDF membrane. The PVDF membrane was placed in a TBST sealant containing 5% skim milk powder for 1 h; then, the primary antibody was added and the membrane was shaken overnight at 4°C. After washing the film, the secondary antibody was added and incubated at 37°C for 1 h. After washing the film, we add the luminescent solution, press the tablet, expose, and fix the film. Lab Works 4.5 image acquisition and analysis system software was used to determine the integrated optical density of protein bands, and GAPDH protein bands were detected simultaneously for each sample as internal reference. Relative expression levels of protein were quantified by integrated gray values analyzed using ImageJ2x software and the bands normalized with the internal reference *β*-actin.

### 2.6. RT-PCR Assay

By Total RNApure Kit (ZOman Biotechnology, Beijing, China), total RNA was prepared. The PrimeScriptTM RT reagent kit(Thermo, USA) was used to generate the cDNA. The reaction conditions were: 42°C for 60 minutes and 70°C for 5 minutes. The cDNA was amplified using a 2x SYBR Green qPCR Master Mix (Thermo, USA). The reaction conditions were 94°C for 3 minutes, 94°C for 30 seconds, and 58°C for 30 seconds for 35 cycles, followed by 72°C for 45 seconds and analysis by a 7500 Thermocycler. The primers are listed in Table 1. The relative differences in gene expression levels of target genes were calculated using the 2^−ΔΔCT^ method.

### 2.7. Statistical Analysis

The data were presented as mean ± SD for at least three different determinations. Statistical analysis was performed with the SPSS version 21. The data were analyzed by GraphPad Prism software, Image J software, and Adobe Photoshop CS6 (San Jose, CA, USA). Differences between variants were analyzed by the Student's *t* test or one-way ANOVA. ^*∗*^*P* < 0.05 ^*∗∗*^*P* < 0.01 were considered statistically significant.

## 3. Results

### 3.1. Expression of STAT1 and STAT3 in Different Lung Adenocarcinoma Cells

**Figure 1:**
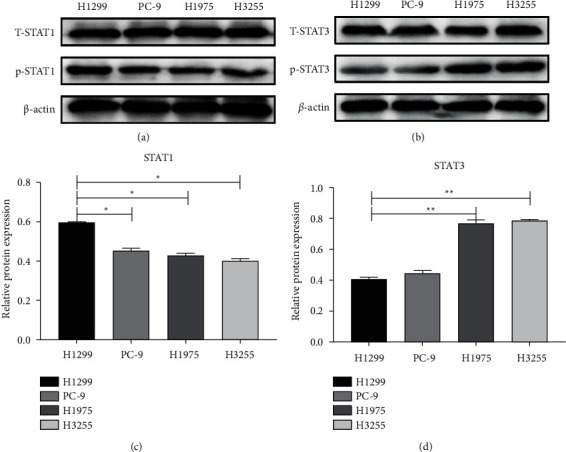
Expression of STAT1 and STAT3 in different lung adenocarcinoma cells. (a) The different levels of the p-STAT1 expression in NSCLC cells (H1299, H1975, PC9, H3255) by western blot. (b) The different levels of p-STAT3 expression in NSCLC cells (H1299, H1975, PC9 and H3255) by western blot. (c) The protein expression level of STAT1 in NSCLC cells (H1299, H1975, PC9 and H3255). (d) The protein expression level of STAT3 in NSCLC cells (H1299, H1975, PC9, and H3255). (^*∗*^*P* < 0.05^*∗∗*^*P* < 0.01).

STATs have emerged as a double-edged sword to some extent, being widely explored in the cancer category. Here, detected the phosphorylation levels of STAT1 and STAT3 in four different lung adenocarcinoma cells and found that the phosphorylation level of STAT1 in H1299 (EGFR-WT) cells was higher than that of PC-9 (EGFR-19del), H1975 (EGFR-T790M and L858R combined mutation), and H3255 (EGFR-L858R mutation) cells (Figure 1(a)), while the phosphorylation level of STAT3 was lower than that of the other three cells (Figure 1(b)). In other words, the phosphorylation level of STAT1 in EGFR wild-type cells was higher than that in EGFR mutant cells (Figure 1(c)), while the phosphorylation level of STAT3 was lower than that in EGFR mutant cells (Figure 1(d)). Therefore, we hypothesized that STAT1 and STAT3 have some crossover mechanism in EGFR wild-type and mutant cells.

## 4. The Biological Regulation of IFN*γ* on Different Types of NSCLC Cells

**Figure 2:**
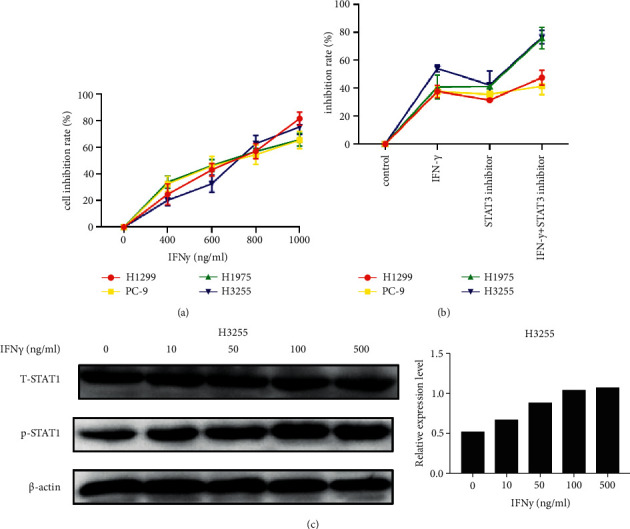
The biological regulation of IFN*γ* on different types of NSCLC cells. (a) Cell viability analysis by CCK assay in NSCLC cell lines treated with a dose gradient of IFN*γ* for 24 h. (b) Cell viability analysis by CCK assay in NSCLC cell lines treated with or without IFN*γ*/STAT3 inhibitor for 24 h. (c) The protein expression level of p-STAT1 detected by western blotting in H3255 treated with different concentrations of IFN*γ* (0, 10, 50, 100, 500 ng/ml).

IFN*γ* is commonly regarded as an inflammatory cytokine which plays a central role in antitumor immunity. Different concentrations of IFN*γ* may produce different biological effects. Different sources of NSCLC cells have different tolerance to IFN*γ*. We demonstrated the effects of IFN*γ* treatment with different concentrations (0, 400, 600, 800, and 1000 ng/ml) on cell viability of H1299 PC-9, H1975, and H3255 by the CCK assay (Figure 2(a)). The results indicated that with the increase of concentration, inhibition of cell proliferation also increased gradually, and were very similar regardless of the cell type. Next, we grouped by with or without IFN*γ* (600 ng/ml)/STAT3 inhibitor and detected the cell viability by the CCK assay (Figure 2(b)). The difference in H3255 was greater in each group than in the other three. The results revealed that there may be some relationship between the IFN*γ*/JAK/STAT1 pathway and JAK/STAT3 pathway. To confirm the correlation between IFN*γ* and STAT1, the expression levels of p-STAT1 in H3255 were detected by western blotting (Figure 2(c)). Results suggested that with the increase of IFN concentration, the phosphorylation level of STAT1 gradually increased and maximized. Based on these results, we determined that the optimal IFN concentration for activation of the JAK/STAT1 signalling pathway to be around 100 ng/ml.

### 4.1. Activation of the IFN*γ*/JAK/STAT1 Pathway Induces Cycle Arrest and Promotes Apoptosis

**Figure 3:**
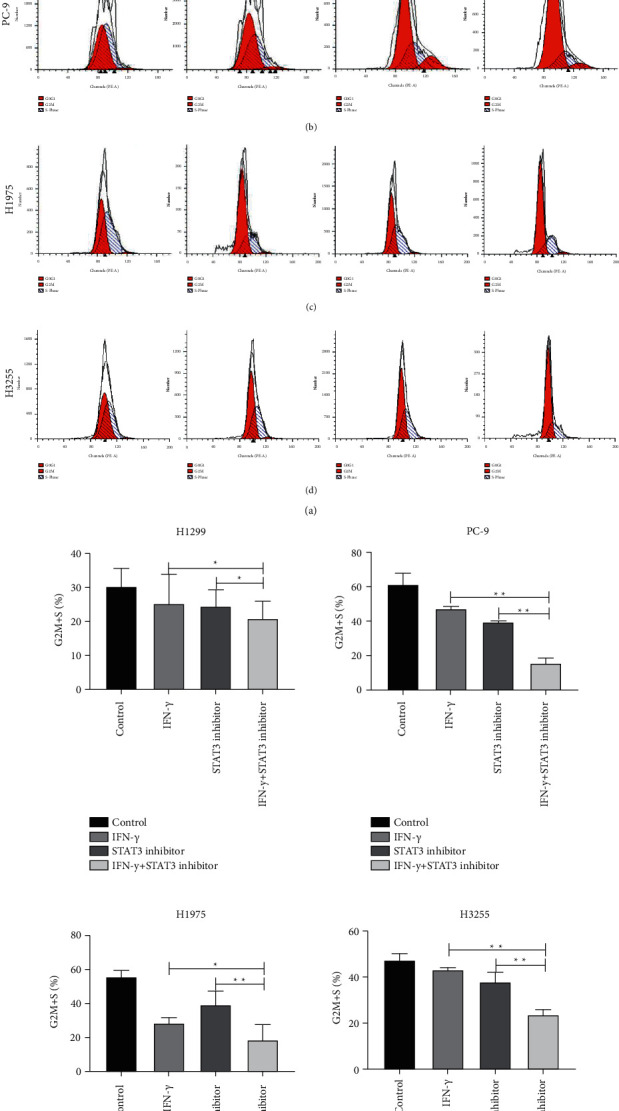
Activation the IFN*γ*/JAK/STAT1 pathway can inhibit NSCLC cell proliferation by inducing cell cycle arrest. (a)–(d) Cell cycle analysis of NSCLC cells (H1299, PC-9, H1975 and H3255) treated with or without IFN*γ*/STAT3 inhibitor by flow cytometry. All data was illustrated as mean ± SD and the asterisks show significant difference as compared with the IFN*γ* group or STAT3 inhibitor group shown by a horizontal line (^*∗*^*P* < 0.05^*∗∗*^*P* < 0.01).

**Figure 4:**
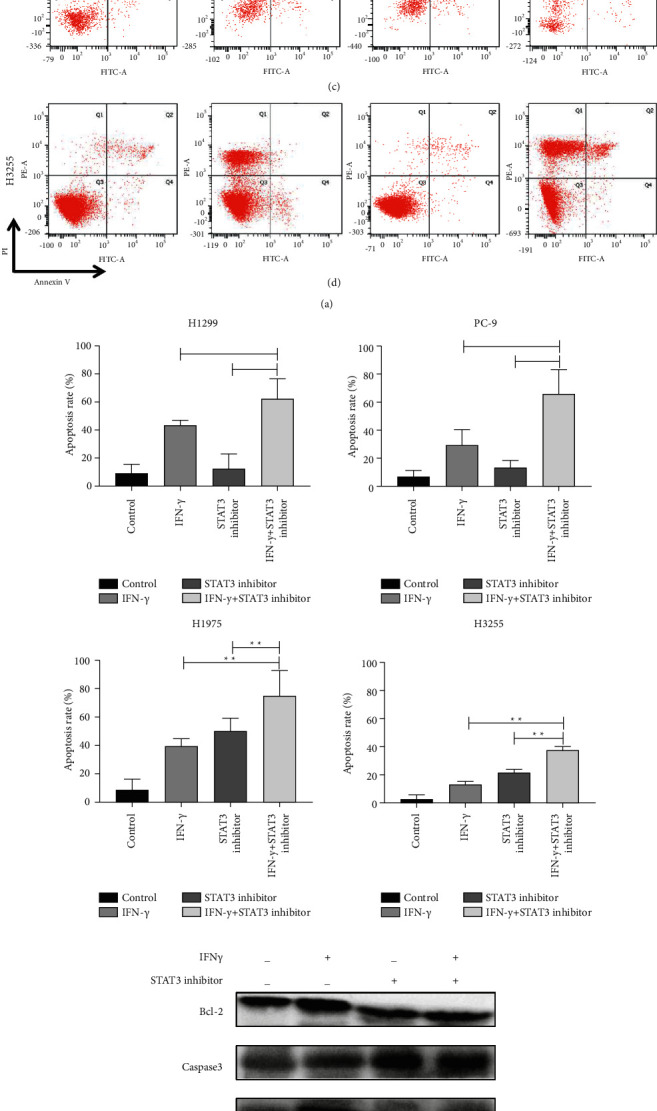
Activation the IFN*γ*/JAK/STAT1 pathway can promote apoptosis. (a)–(d) Apoptosis analysis of NSCLC cells (H1299, H1975, PC9 and H3255) treated combined with or without IFN*γ*/STAT3 inhibitor by Annexin-V/PI and flow cytometry. (e) Immunoblotting analysis of apoptin expression in H3255 cells treated with or without IFN*γ*/STAT3 inhibitor. All data were illustrated as mean ± SD (^*∗*^*P* < 0.05^*∗∗*^*P* < 0.01).

To figure out whether cell cycle distribution and apoptosis were altered by inhibition of STAT3 phosphorylation in NSCLC cells, we conducted flow cytometric assays. The analysis of flow cytometry showed that H1299, PC-9, H1975, and H3255 cells treated with a combination of IFN*γ* and STAT3 inhibitor had higher rates of G2M + S cell cycle arrest compared to treatment with IFN*γ* or STAT3 inhibitor alone (Figures 3(a)–3(d)). In order to further confirm whether it was inhibition of STAT3 phosphorylation to further promote cell apoptosis, STAT3 inhibitors were used to combination IFN*γ* and flow cytometry analysis was performed (Figures 4(a)–4(d)). As shown in Figure 4, inhibition of STAT3 phosphorylation and IFN*γ* stimulation can further increase the apoptosis rate. Whereas the high level of IFN*γ* activated the JAK1-STAT1-caspase pathway and then induced apoptosis in NSCLC. The detection of apoptotic markers in protein expression levels also verified that inhibition of STAT3 phosphorylation and IFN*γ* stimulation decreased H3255 cells by increasing the expression of caspase-3/Bax and suppressing the level of Bcl-2 (Figure 4(e)). Taken together, these data indicated that combination with IFN*γ* and STAT3 inhibitor could inhibit the proliferation of lung cancer cells by promoting apoptosis and cell cycle transition. Based on the above data, we speculate that there may be antagonistic effects between the IFN/JAK/STAT1 and JAK/STAT3 signalling pathways.

### 4.2. Inhibition of STAT3 Phosphorylation Further Activates the IFN*γ*/JAK/STAT1 Signalling Pathway

**Figure 5:**
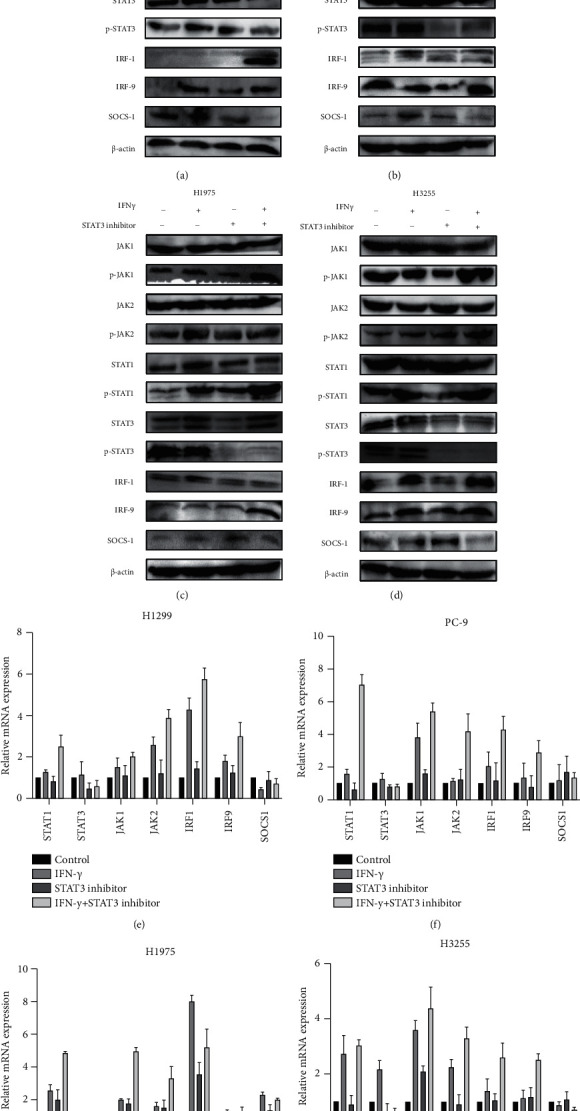
Inhibition of STAT3 phosphorylation further activates the IFN*γ*/JAK/STAT1 signalling pathway. (a)–(d) The relative proteins expressions of difference groups in NSCLC cells (H1299, PC-9, H1975 and H3255) by western blotting assay. (e)–(h) qRT-PCR was used to analyse the mRNA expression levels of relative genes in NSCLC cells (H1299, H1975, PC9, H3255) treated combined with or without IFN*γ*/STAT3 inhibitor. All data were illustrated as mean ± SD and the asterisks show significant difference.

To confirm the antagonistic effects between IFN*γ*/JAK/STAT1 and JAK/STAT3 pathways, expressions of key molecules were analysed by western blot and qRT-PCR. Compared to the other three groups, every protein level and mRNA level of phosph-STAT1, the targets expression levels in the IFN*γ* and STAT3 inhibitor combination group were the highest in NSCLC cells (H1299, H1975, PC9, and H3255) (Figures 5(a)–5(d)), suggesting that inhibition of STAT3 phosphorylation may further activate the STAT1 phosphorylation level. In addition, IFN*γ*/JAK/STAT1 signal transduction pathway genes, such as interferon regulator 1 (IRF-1) and IRF-9, are directly induced by IFN*γ* through the JAK/STAT1 signal transduction pathway. SOCS1 inhibits the IFN*γ* signalling pathway and T cell differentiation, possibly by a mechanism whereby SOCS1 specifically inhibits gp130 and STAT1 phosphorylation. Cells without STAT1 gene cannot be induced to produce SOCS1 due to IFN*γ* signalling pathway obstruction, which may induce malignant cell proliferation, thus SOCS1 is also regarded as a tumor inhibitor. Therefore, compared to the other three groups, every protein level and mRNA level, the targets of IRF1 and IRF9 expression levels in the IFN*γ* and STAT3 inhibitor combination group were the highest and the SOCS1 lowest in NSCLC cells (H1299, H1975, PC9, and H3255) (Figures 5(e)–5(h)), suggesting the inhibition of STAT3 phosphorylation could further activates the IFN*γ*/JAK/STAT1 signalling pathway. Data indicated that the role of competitive inhibition in the IFN*γ*/JAK/STAT1 and JAK/STAT3 signalling pathways.

### 4.3. Inhibition of STAT3 Phosphorylation Further Induced the PD-L1 Expression

**Figure 6:**
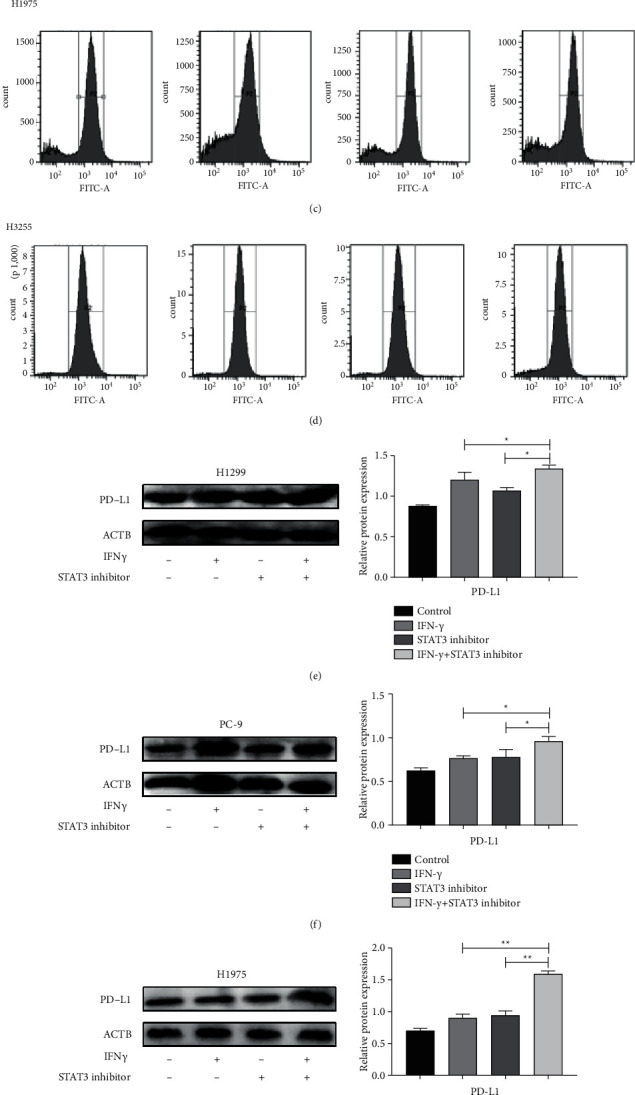
Inhibition of STAT3 phosphorylation further induced PD-L1 expression. (a)–(d) The different levels of PD-L1 expression in NSCLC cells (H1299, H1975, PC9 and H3255) treated with or without IFN*γ*/STAT3 inhibitor by flow cytometry. (e)–(h) The protein expression level of PD-L1 in NSCLC cells (H1299, H1975, PC9 and H3255) treated with or without IFN*γ*/STAT3 inhibitor. All data was illustrated as mean ± SD (^*∗*^*P* < 0.05^*∗∗*^*P* < 0.01).

In the early stages of our study, the application of chemotherapy or EGFR-TKIs for EGFR mutation patients was found to be an increase in the expression of PD-L1 in the tumor cell surface, and the PD-L1 was significantly reduced after the treatment of the anti-PD-1. IFN*γ* could induce PD-L1 expression via IFN*γ*/JAK/STAT1 signalling pathways. To further confirm the antagonism between the IFN*γ*/JAK/STAT1 and JAK/STAT3 signalling pathways, we recorded the different levels of PD-L1 expression in NSCLC cell lines treated with or without IFN*γ*/STAT3 inhibitor by flow cytometry (Figures 6(a)–6(d)). Results displayed the protein level of PD-L1 in the IFN*γ* and STAT3 inhibitor combination group was significantly higher than that in the others. By western blot of cell lines, we found that the expression of PD-L1 in the IFN*γ* and STAT3 inhibitor combination group was significantly higher than the other three groups (Figures 6(e)–6(h)), which was consistent with the results of flow cytometry. These results confirm that inhibition of STAT3 phosphorylation further activates the IFN*γ*/JAK/STAT1 signalling pathway.

## 5. Discussion

In recent years, immune checkpoint blockers have brought new hope to NSCLC treatment with a new mechanism of action and effect advantage [20]. Unlike traditional antitumor drugs, immunotherapy kills tumor cells by activating the body's immune cells, bringing long-term survival benefits, and lasting immune responses to patients. Multiple ICI clinical trials have shown that once effective, other therapies will have a longer life span. In the recently reported “NADIM” study of ICIs for the neoadjuvant therapy of NSCLC, the results were even more promising, with a disease control rate of 96%, showing once again the great application prospect and therapeutic potential of immunotherapy [21]. However, EGFR mutation was once considered as a marker of immune resistance, but the mechanism is difficult to determine [13]. In recent years, several clinical studies have brought new enlightenment: the so-called “primary resistance” of EGFR mutation-positive NSCLC patients to ICIs is not absolute [22]. After TKI resistance, some studies on double immunization or immunocombined chemotherapy have achieved initial results, and ICIs combined with chemotherapy and antivascular therapy have considerable efficacy [23–25]. Moreover, in vitro studies have shown that EGFR mutation-positive NSCLC can change its non-inflammatory tumor microenvironment and improve its responsiveness to PD1 inhibitors after intervention [26]. Therefore, it is of great importance to further study the characteristics of the immune microenvironment of EGFR mutation-positive NSCLC and the mechanism of primary ICIs resistance and explore ways to reverse drug resistance and new treatment modes, which will hopefully open up the way for immunotherapy of EGFR mutation-positive NSCLC patients.

In early in vivo studies, we found that chemotherapy or EGFR-TKIs treatment for patients with EGFR mutations could cause the expression of PD-L1 on the surface of tumor cells to be upregulated, while the expression of PD-L1 was significantly downregulated after anti-PD-1 monoclonal antibody treatment [27]. Furthermore, we found that further inhibition of the PD-L1 expression at the cellular level could cause changes in a series of tumor microenvironmental cytokines represented by tumor-related macrophages, as well as the apoptosis of lung adenocarcinoma cells, which preliminarily confirmed the close relationship among the signalling pathway promoting tumor growth and the immune microenvironment and immune efficacy [28]. Therefore, it is urgent to further study the possible mechanism of primary immune resistance in EGFR mutant population and explore the mechanism of reverse resistance. This is expected to provide a more solid theoretical basis for the application of back-line immunotherapy in patients with EGFR mutations.

**Figure 7:**
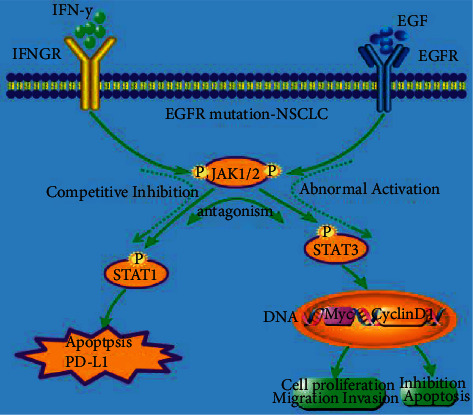
Schematic illustration of the interaction between the IFN*γ*/JAK/STAT1 and JAK/STAT3 signalling pathways. The abnormal activation of the downstream JAK/STAT3 pathway in the EGFR mutation cell may have antagonistic effects on the STAT1 induced anti-tumor immune response, which may cause the IFN*γ*-JAK-STAT1 pathway to lose its function, resulting in the production of the immune tolerance of the EGFR mutant, which promotes immune escape. Summary diagram of molecular mechanism of the interaction between the IFN*γ*/JAK/STAT1 and JAK/STAT3 signalling pathways in lung cancer cells.

Previous studies demonstrated that IFN signalling pathway loss is one of the main mechanisms leading to endogenous drug resistance of ICIs [29]. Therefore, mutations and deletions of proteins associated with this pathway on tumor cells, such as JAK1 and JAK2, STAT1, IRF1, and other IFN receptor chains, can lead to drug resistance to immunocheckpoint inhibitors [17]. EGFR mutations cause abnormal activation of the downstream JAK/STAT3 signalling pathway, leading to increased expression of phosphorylated STAT3, followed by abnormal tumor growth, angiogenesis, invasion, and metastasis. Existing studies have confirmed that the STAT3-mediated inhibitory tumor immune response pathway has an antagonistic effect on the STAT1-driven antitumor immune response, which may lead to the loss of IFN*γ*/JAK/STAT1 pathway function (Figure 7).

In the present study, we used CCK assay and flow cytometry analysis and these data indicated that combination with IFN*γ* and STAT3 inhibitor could promote apoptosis and inhibit cell cycle transition to inhibit the proliferation of lung cancer cells. Based on the above data, we speculate that there may be antagonistic effects between the IFN*γ*/JAK/STAT1 and JAK/STAT3 signalling pathways. Next, the western blot and qRT-PCR results revealed that compared to the other three groups, protein level and mRNA level of phosph-STAT1, the target in the IFN*γ* and STAT3 inhibitor combination group were the highest in NSCLC cells (H1299, H1975, PC9, H3255). Furthermore, the targets of IRF1 and IRF9 expression levels in the IFN*γ* and STAT3 inhibitor combination group were the highest and the SOCS1 lowest in NSCLC cells (H1299, H1975, PC9, and H3255). This data indicated that the role of competitive inhibition in the IFN*γ*/JAK/STAT1 and JAK/STAT3 signalling pathways.

In conclusion, our findings showed that Inhibition the STAT3 phosphorylation could further activate the IFN*γ*/JAK/STAT1 signalling pathway in NSCLC cells. The role of competitive inhibition in the IFN*γ*/JAK/STAT1 and JAK/STAT3 signalling pathways. Our study could provide a novel therapeutic strategy for immunotherapy in NSCLC patients with EGFR mutation positive and immunoresistance. It also provides a theoretical basis for future combination of STAT3 inhibitors and immunecheckpoint inhibitors.

## Figures and Tables

**Table 1:** Primer sequences of related genes for RT-qPCR.

Genes	Sequence (5′–3′)
STAT1	Forward: 5′-AACCTCGACAGTCTTGGCAC-3′
	Reverse: 5′-AGACATCCTGCCACCTTGTG-3′
STAT3	Forward: 5′-TCCTGAAGCTGACCCAGGTA-3′
	Reverse: 5′-TATTGCTGCAGGTCGTTGGT-3′
JAK1	Forward: 5′-GCATCGAGCGCACAAAGTTA-3′
	Reverse: 5′-ACCAGTAGGGTTGAGGGACA-3′
JAK2	Forward: 5′-CTGAGTTCGAAGCTAGCAGGG-3′
	Reverse: 5′-CCCGTCACAGTTGTCTCCAC-3′
IRF1	Forward: 5′-AAAGTCGAAGTCCAGCCGAG-3′
	Reverse: 5′-TGTTGTAGCTGGAGTCAGGG-3′
IRF9	Forward: 5′-ACCAGGATGCTGCCTTCTTC-3′
	Reverse: 5′-CCTGGTGGCAGCAACTGATA-3′
SOCS1	Forward: 5′-CACTTCCGCACATTCCGTTC-3′
	Reverse: 5′-AGGCCATCTTCACGCTAAGG-3′

## Data Availability

The data that support the findings of this study are available from the corresponding author upon reasonable request.
